# Bilateral Roth spots following metapneumovirus infection: A rare but underrecognized association

**DOI:** 10.1016/j.ajoc.2026.102642

**Published:** 2026-08-07

**Authors:** Thomas Foulonneau, Tali Anne Szwebel, Rebecca Farhat, Deborah Eshagh, Antoine P. Brezin, Dominique Monnet

**Affiliations:** a Service d'ophtalmologie, AP-HP, Hôpital Cochin, Paris, 75014, France; b Service de Médecine Interne, AP-HP, Hôpital Cochin, Paris, 75014, France

**Keywords:** Roth spots, Viral infection, Retinal hemorrhages, Metapneumovirus

## Abstract

**Purpose:**

To report a case of bilateral Roth spots in an adolescent, with a recent metapneumovirus viral infection.

**Observations:**

A 17-year-old male consulted with painless floaters and blurred vision. Fundus examination showed bilateral Roth spots and segmental venous sheathing. An extensive systemic workup was unremarkable, including negative QuantiFERON-TB Gold testing. The patient had a recent history of viral infection, and a metapneumovirus was detected on PCR of a nasal swab.

**The retinal lesions resolved spontaneously:**

by day 7, visual acuity was normal and Goldmann visual field testing showed no residual defect. At the one-year follow-up, complete resolution was sustained on both color fundus photography and fundus autofluorescence, with no residual changes of the retinal pigment epithelium.

**Conclusion and importance:**

This case highlights a rare association between a viral respiratory infection and Roth spots. The lesions are presumed to be due to a post-viral immune-mediated process affecting the retinal microvasculature. The rapid spontaneous resolution and the absence of long-term sequelae suggest that similar cases may go unnoticed.

## Highlights

• Bilateral Roth spots were observed in a healthy adolescent after a metapneumovirus infection.
• The retinal lesions resolved spontaneously, with sustained resolution at one-year follow-up.
• The lesions are presumed to be linked to a virus-induced immune-mediated process affecting retinal microvasculature.

## 1 Introduction

Roth spots are retinal hemorrhages with a white center composed of fibrin and inflammatory cells. They are classically linked to subacute endocarditis, leukemia, and severe anemia, but can also occur in inflammatory and infectious conditions.^1^ Although viral infections have been associated with retinal hemorrhages, their relationship with Roth spots remains uncommon and is typically observed in the setting of virus-induced hematologic abnormalities, such as anemia or thrombocytopenia.^2^

In the context of respiratory viruses, this association appears to be particularly rare. After conducting a literature review on May 30, 2025, utilizing PubMed, Google Scholar, and Embase with the keywords “Roth spots,” “metapneumovirus,” and “retinal hemorrhages,” we did not find any prior reports of bilateral Roth spots following a metapneumovirus infection.

## 2 Case report

A 17-year-old male with no significant past medical history presented with bilateral floaters and blurred vision persisting for five days. There was no associated pain or photophobia. The patient had recently traveled to the United Kingdom and reported an upper respiratory infection one week before the onset of the ocular manifestations.

**Fig. 1:**
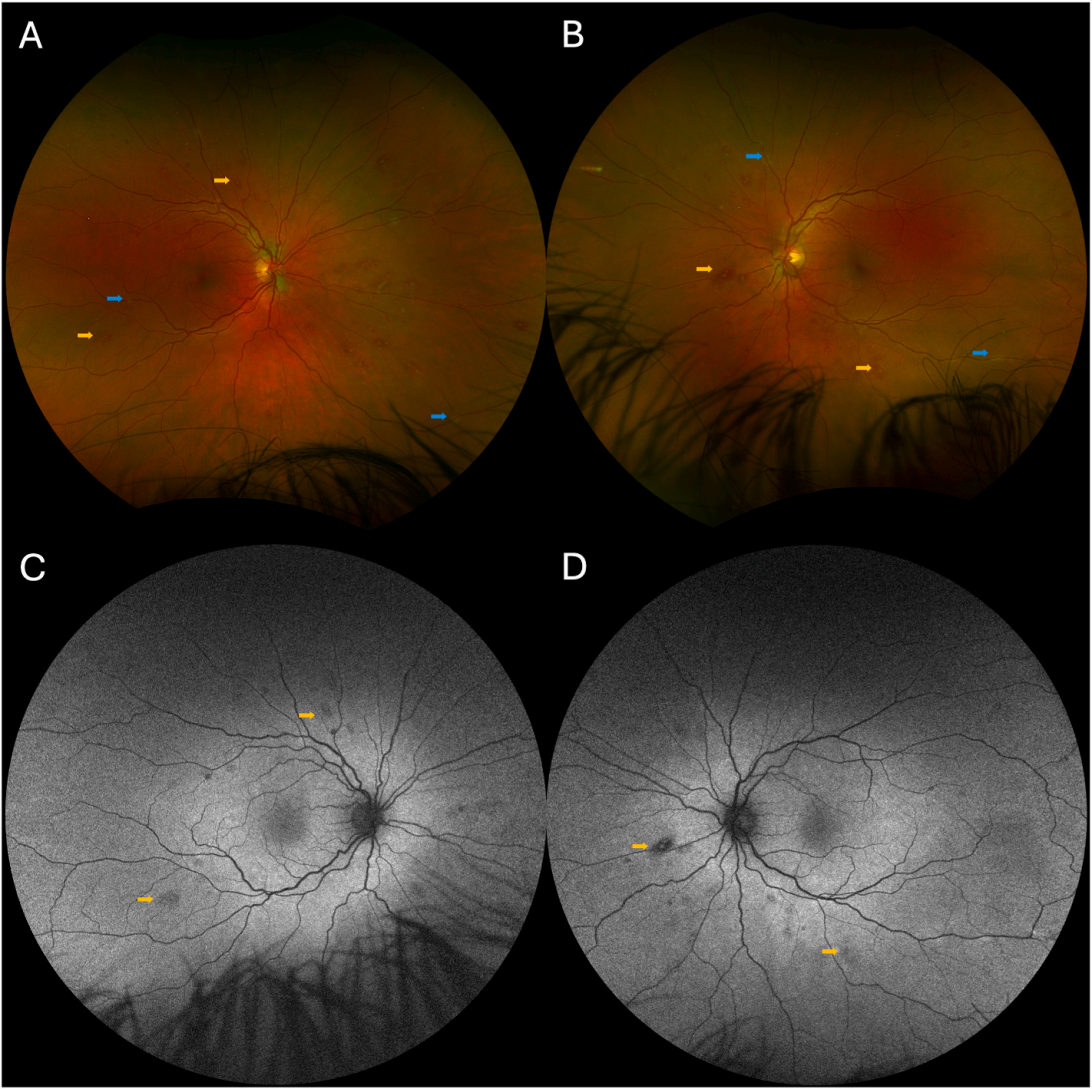
Baseline morphological imaging of both eyes at presentation. (A–B) Ultra-widefield color fundus photographs of the right (A) and left (B) eye showing bilateral Roth spots (yellow arrows) and segmental peripapillary venous sheathing (cyan arrows). (C–D) Fundus autofluorescence of the right (C) and left (D) eye showing hypoautofluorescent lesions (yellow arrows) corresponding to the Roth spots seen on color photography. (For interpretation of the references to color in this figure legend, the reader is referred to the Web version of this article.)

The best corrected visual acuity was 20/20 in both eyes. The intraocular pressure was normal in both eyes. The anterior segment examination revealed trace anterior chamber cells, without vitritis. The dilated fundus examination showed bilateral Roth spots with segmental venous sheathing, without choroidal involvement (Fig. 1A and B).

**Fig. 2:**
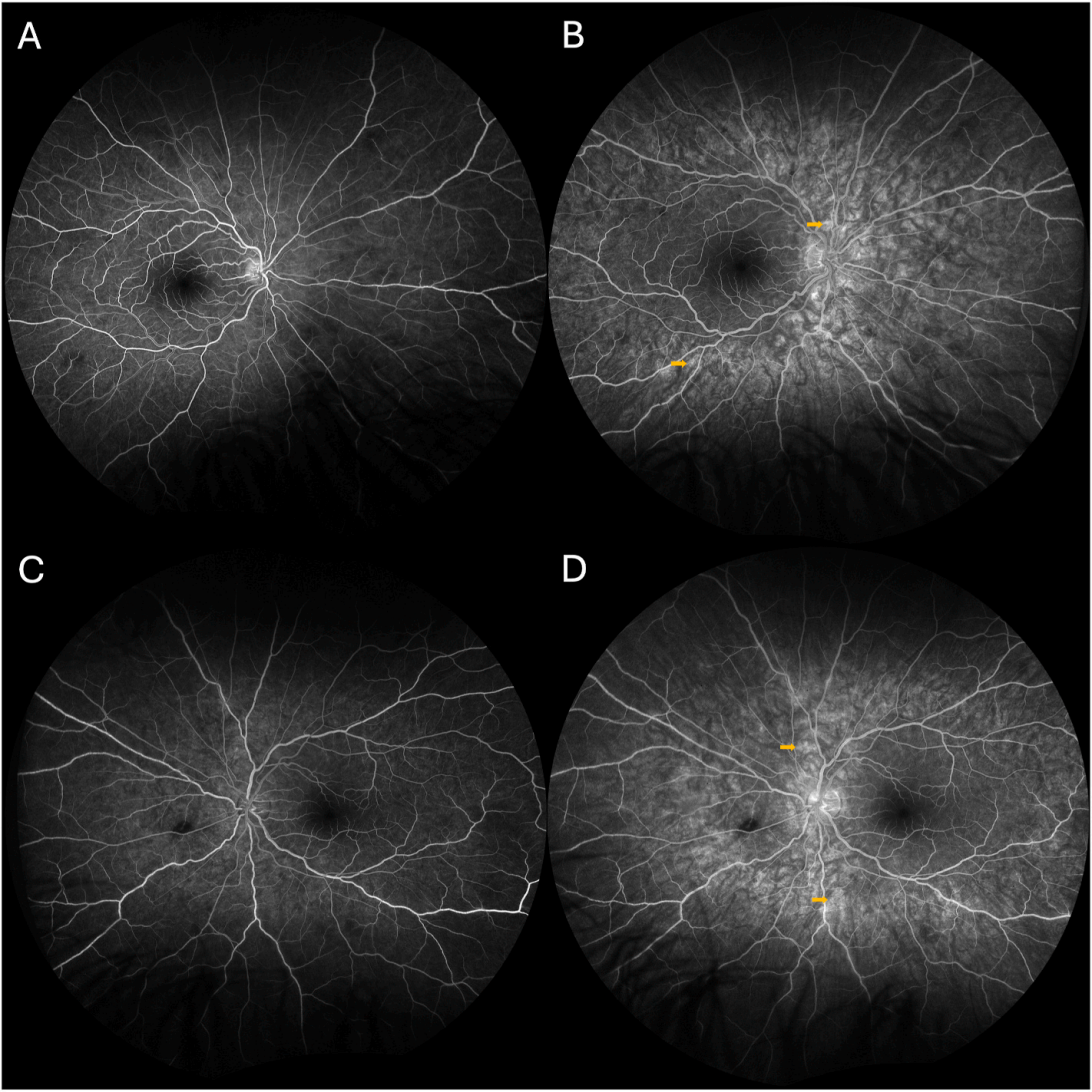
Baseline ultra-widefield fluorescein angiography (Optos) of both eyes. (A–B) Right eye at 1 min (A) and 5 min (B); (C–D) Left eye at 1 min (C) and 5 min (D). The mid- and late-phase frames demonstrate diffuse posterior-pole granular capillary leakage with peripapillary venous wall staining (yellow arrows), without focal active vasculitis or peripheral capillary non-perfusion, consistent with a diffuse retinal microvascular involvement rather than an occlusive vasculitis. (For interpretation of the references to color in this figure legend, the reader is referred to the Web version of this article.)

**Fig. 3:**
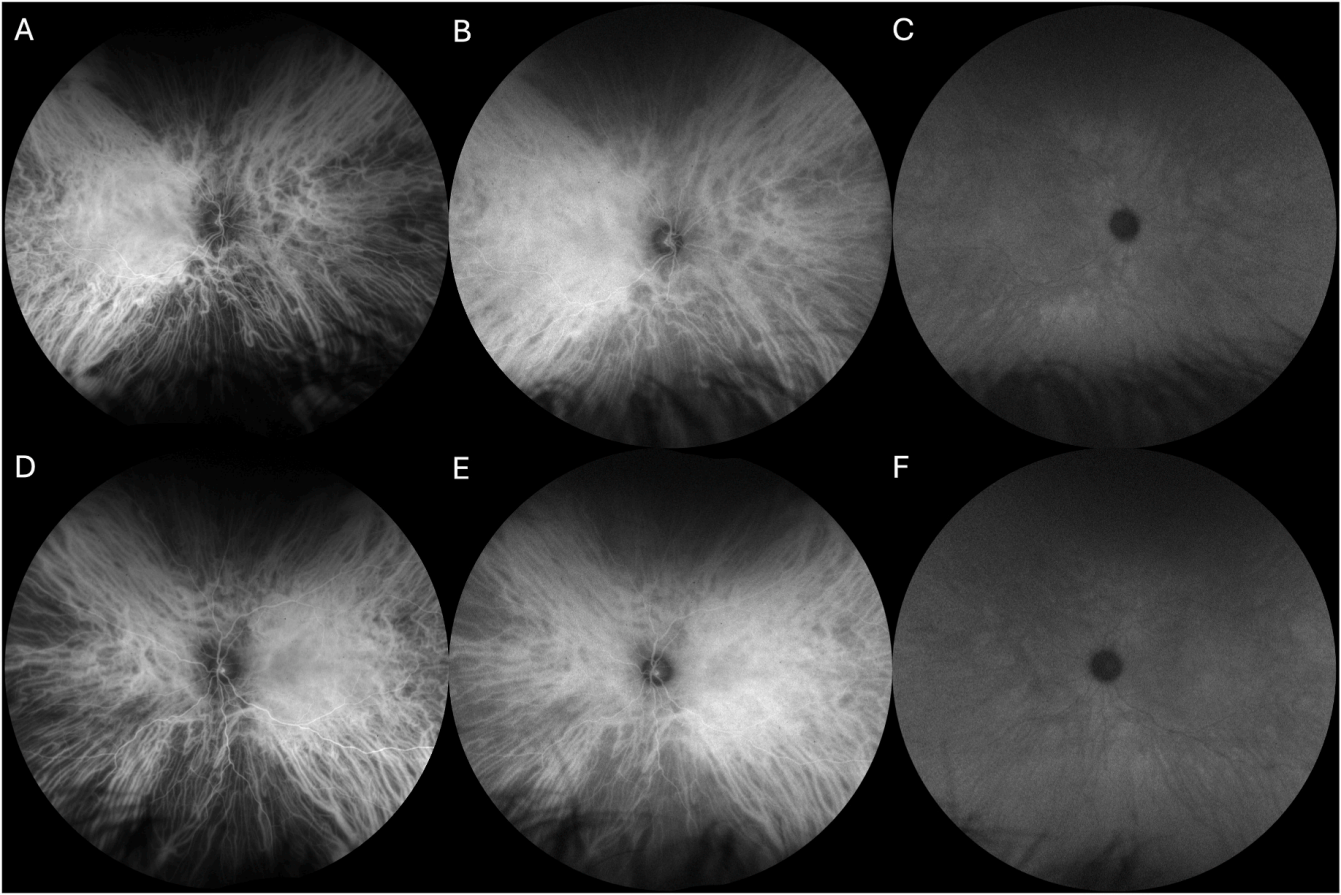
Baseline indocyanine green (ICG) angiography of both eyes at presentation. (A–C) Right eye at 1 min (A), 5 min (B), and 15 min (C); (D–F) Left eye at 1 min (D), 5 min (E), and 15 min (F). The early, mid-phase, and late-phase frames demonstrate no choroidal hypocyanescent dots, no late staining, and no focal choroidal involvement, ruling out an associated choroiditis.

Multimodal imaging confirmed these findings. Fundus autofluorescence revealed hypoautofluorescent lesions corresponding to the Roth spots (Fig. 1C and D). Ultra-widefield fluorescein angiography (Optos) acquired at 1 min and 5 min (Fig. 2) demonstrated diffuse posterior-pole capillary leakage with peripapillary venous wall staining, without focal active vasculitis, and importantly showed no peripheral capillary non-perfusion or vascular occlusion, arguing against an occlusive vasculitis. Indocyanine green (ICG) angiography acquired at one, five, and 15 min (Fig. 3), including the late-phase frames, was unremarkable, with no choroidal hypocyanescent dots, no late staining, and no focal choroidal involvement.

Extensive laboratory testing ruled out infectious, autoimmune, and hematologic causes. The complete blood count and inflammatory markers were normal. Serologies for HIV, syphilis, hepatitis B/C, and Bartonella were negative, and QuantiFERON-TB Gold testing was negative. Autoimmune screening, including ANA, ANCA, and complement levels, was unremarkable. Echocardiography showed no valvular vegetations. Lumbar puncture and chest CT-scan were normal. A multiplex PCR of nasal swabs was positive for metapneumovirus. Anterior chamber paracentesis with aqueous humor analysis was not performed, given the preserved 20/20 visual acuity, the presence of only trace anterior chamber cells, the absence of vitritis, and the rapid spontaneous resolution of the retinal findings.

**Fig. 4:**
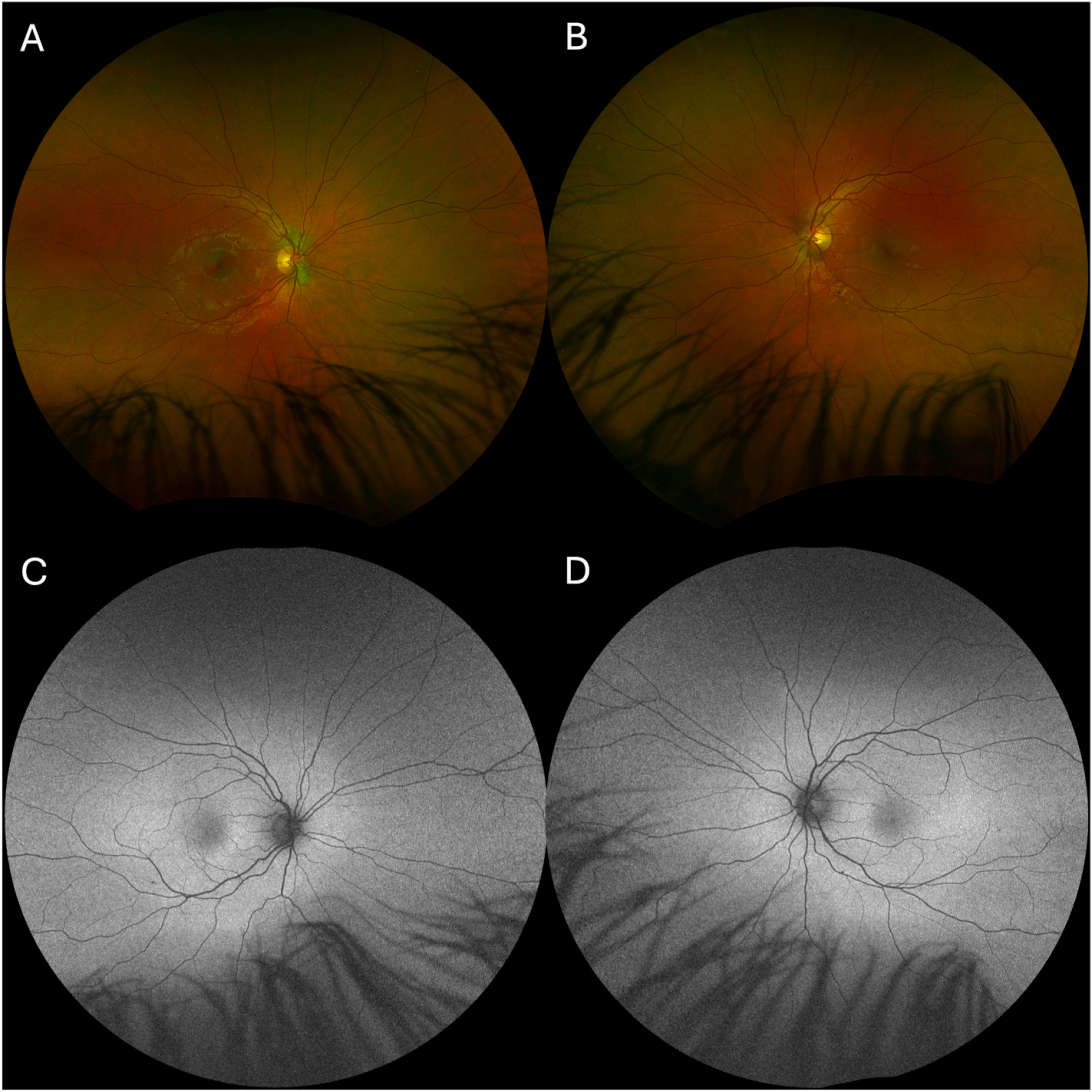
One-year follow-up imaging of both eyes. (A–B) Ultra-widefield color fundus photographs of the right (A) and left (B) eye showing complete and sustained resolution of the bilateral Roth spots and venous sheathing. (C–D) Fundus autofluorescence of the right (C) and left (D) eye showing no residual hypoautofluorescent lesions and no abnormality of the retinal pigment epithelium. (For interpretation of the references to color in this figure legend, the reader is referred to the Web version of this article.)

The patient was monitored without systemic treatment. By day 7, the bilateral Roth spots and the segmental venous sheathing had completely resolved on ultra-widefield color fundus photography, best corrected visual acuity remained 20/20 in both eyes, and Goldmann visual field testing was normal. Repeat fluorescein angiography was not performed given the complete clinical and photographic resolution and the absence of any visual symptom. At the one-year follow-up, sustained complete resolution was observed on both ultra-widefield color fundus photography and fundus autofluorescence, with no residual changes of the retinal pigment epithelium (Fig. 4).

## 3 Discussion

Roth spots are often associated with subacute bacterial endocarditis but can also result from systemic diseases. Previous reports have linked viral infections such as Epstein-Barr virus and Human immunodeficiency virus to retinal hemorrhages.^3^ In this case, the temporal relationship with metapneumovirus suggested a viral-induced immune response affecting the retinal microvasculature.

The pattern of imaging findings observed in our patient is consistent with a primary retinal microvascular involvement rather than an occlusive vasculitis: ultra-widefield fluorescein angiography demonstrated diffuse posterior-pole capillary leakage with peripapillary venous wall staining, but without peripheral capillary non-perfusion or vascular occlusion. The serial ICG angiography, including late-phase frames, excluded any associated choroidal involvement. The complete and durable resolution of both the clinical and imaging findings at one year, with no autofluorescence abnormality of the retinal pigment epithelium, further supports a self-limited, non-destructive post-viral immune-mediated process.

Several limitations of our report should be acknowledged. Repeat fluorescein angiography was not performed at follow-up because the patient was fully asymptomatic with complete photographic and autofluorescence resolution of the retinal lesions; angiographic confirmation of the resolution of the diffuse capillary leakage is therefore lacking. In addition, intraocular sampling (anterior chamber paracentesis with aqueous humor PCR) was not performed, as the clinical context — preserved visual acuity, minimal anterior chamber inflammation, and rapid spontaneous resolution — did not justify an invasive procedure. The causal relationship between the metapneumovirus infection and the retinal findings therefore remains presumptive, based on the temporal association and on the exclusion of alternative etiologies.

## 4 Conclusion

This case illustrates a self-limited form of bilateral Roth spots potentially related to a recent metapneumovirus viral infection, with sustained complete resolution at one-year follow-up. While viral infections have been previously implicated in retinal hemorrhages, their role in Roth spots remains underrecognized and warrants further investigation.

## Patient consent

Written consent to publish this case has not been obtained. This report does not contain any personal identifying information.

## Claim of priority

After conducting a literature review on May 30, 2025, utilizing PubMed, Google Scholar, and Embase using the key words “Roth spots,” “metapneumovirus,” and “retinal hemorrhages,” we did not find any prior reports of bilateral Roth spots following a metapneumovirus infection.

## Funding

No funding or grant support.

## CRediT authorship contribution statement

**Thomas Foulonneau:** Investigation, Writing – original draft. **Tali Anne Szwebel:** Investigation. **Rebecca Farhat:** Investigation. **Deborah Eshagh:** Investigation. **Antoine P. Brezin:** Investigation. **Dominique Monnet:** Investigation, Supervision, Writing – review & editing.

## Declaration of competing interest

The authors declare that they have no known competing financial interests or personal relationships that could have appeared to influence the work reported in this paper.
